# *Trichoderma atroviride* from Predator to Prey: Role of the Mitogen-Activated Protein Kinase Tmk3 in Fungal Chemical Defense against Fungivory by *Drosophila melanogaster* Larvae

**DOI:** 10.1128/AEM.01825-18

**Published:** 2019-01-09

**Authors:** Karina Atriztán-Hernández, Abigail Moreno-Pedraza, Robert Winkler, Therese Markow, Alfredo Herrera-Estrella

**Affiliations:** a National Laboratory of Genomics for Biodiversity, CINVESTAV, Irapuato, Guanajuato, México; b Department of Biotechnology and Biochemistry, Unidad Irapuato, CINVESTAV, Irapuato, Guanajuato, México; c Section of Cell and Developmental Biology, Division of Biological Sciences, University of California at San Diego, La Jolla, California, USA; Wageningen University

**Keywords:** Tmk3, *Trichoderma*, chemical response, fungivory, injury, metabolite

## Abstract

The response to injury represents an important strategy for animals and plants to survive mechanical damage and predation. Plants respond to injury by activating a defense response that includes the production of an important variety of compounds that help them withstand predator attack and recover from mechanical injury (MI). Similarly, the filamentous fungus Trichoderma atroviride responds to MI by strongly modifying its transcriptional profile and producing asexual reproduction structures (conidia). Here, we analyzed whether the response to MI in T. atroviride is related to a possible predator defense mechanism from a metabolic perspective. We found that the production of specific groups of secondary metabolites increases in response to MI but is reduced after fungivory by Drosophila melanogaster larvae. We further show that fungivory results in repression of the expression of genes putatively involved in the regulation of secondary metabolite production in T. atroviride. Activation of secondary metabolite production appears to depend on the mitogen-activated protein kinase (MAPK) Tmk3. Interestingly, D. melanogaster larvae preferred to feed on a *tmk3* gene replacement mutant rather than on the wild-type strain. Consumption of the mutant strain, however, resulted in increased larval mortality.

**IMPORTANCE** Fungi, like other organisms, have natural predators, including fungivorous nematodes and arthropods that use them as an important food source. Thus, they require mechanisms to detect and respond to injury. Trichoderma atroviride responds to mycelial injury by rapidly regenerating its hyphae and developing asexual reproduction structures. Whether this injury response is associated with attack by fungivorous insects is unknown. Therefore, determining the possible conservation of a defense mechanism to predation in T. atroviride and plants and elucidating the mechanisms involved in the establishment of this response is of major interest. Here, we describe the chemical response of T. atroviride to mechanical injury and fungivory and the role of a MAPK pathway in the regulation of this response.

## Abstract (precis)

Fungi, like other organisms, have natural predators, including fungivorous nematodes and arthropods that use them as an important food source. Thus, they require mechanisms to detect and respond to injury. Trichoderma atroviride responds to mycelial injury by rapidly regenerating its hyphae and developing asexual reproduction structures. Whether this injury response is associated with attack by fungivorous insects is unknown. Therefore, determining the possible conservation of a defense mechanism to predation in T. atroviride and plants and elucidating the mechanisms involved in the establishment of this response is of major interest. Here, we describe the chemical response of T. atroviride to mechanical injury and fungivory and the role of a MAPK pathway in the regulation of this response.

## INTRODUCTION

In nature, all organisms are constantly exposed to different kinds of abiotic (UV radiation, drought, and nutrient shortage, among others) and biotic (competitors, pathogens, and predators) stresses (1–3). Accordingly, they have developed stress response strategies that have been preserved during their evolutionary history. Such strategies increase their capacity to contend with a variety of environmental challenges (2, 4). Fungi, like plants, are sessile organisms unable to escape attacks by predators, and their spores and hyphae are nutrient sources for fungivorous mites, collembolans, nematodes, and insects (5). Studies in *Aspergillus* spp. (6–8) and Coprinopsis cinerea (9, 10), using Drosophila melanogaster larvae, the collembolans Folsomia candida, and the nematode Aphalenchus avenae as fungivory models (6–9, 11), have provided evidence of a chemical defense by fungi against predators, and the existence of specific recognition has been postulated (9, 10).

The available evidence supporting the establishment of a fungal chemical defense against fungivorous insects has been obtained mostly using *Aspergillus* strains with mutations affecting secondary metabolite production (7, 11–13). Those studies showed that an Aspergillus nidulans strain with a mutation in *laeA*, a gene encoding a putative methyltransferase involved in sterigmatocystin (ST) production, was more susceptible to predation by D. melanogaster larvae than the wild-type (WT) strain (12). Additionally, in food choice assays with a choice of strains overexpressing *rsmA*, with higher production of ST than the WT, the collembolan Folsomia candida preferred to feed on the WT strain (14). The authors thus proposed that ST is important for fungal resistance or defense against predators (7, 11, 13, 14). In this sense, Spiteller described the chemical response to injury in fruiting bodies of basidiomycetes and proposed that fungi, like plants, display three mechanisms of chemical defense (constitutive, wound-activated, and induced chemical defense) (15, 16). The constitutive chemical defense relies on compounds that are permanently produced (toxins, pungent and bitter compounds, and fungicides) (15, 16). During wound-activated chemical defense, inactive precursors of secondary metabolites (SM) are transformed into their biologically active form after injury, producing compounds with antibacterial, antifungal, and toxic activities against insects and mammals (15, 16). While induced chemical defense is based on *de novo* synthesis or increase of a defense compound after activation of genes related to its biosynthesis, such a process could take hours (15, 16). Studies of the defense responses of filamentous fungi to injury are, however, scarce.

Members of the genus *Trichoderma* are cosmopolitan organisms, widely used as biocontrol agents against phytopathogenic organisms. Several species of this genus establish beneficial relationships with plants, promote plant growth, and are considered mycoparasites. Other species are used in industry to produce biofuels, enzymes, and a great variety of metabolites important for different industrial or medical applications (17–19). The antagonistic capacity of *Trichoderma* spp. against bacteria and fungi is, at least in part, explained by their capacity to produce a wide range of bioactive metabolites with antifungal, antimicrobial, and cytotoxic activities (peptaibols, glyotoxins, gliovyridins, pyrones, and terpenes) (20, 21). Although *Trichoderma* spp. are believed to produce more than 1,000 different molecules, depending on the strain and growth conditions, only 120 metabolites have had their structures described. Furthermore, very little is known about the role of these compounds in the biology of *Trichoderma* (20).

Several studies of fungus-insect interactions indicate that, in nature, *Trichoderma* spp. could be exposed to attack by insects. Um and coworkers (22) described the opportunistic ability of *Trichoderma* to antagonize colonies of the fungus *Termitomyces*, a symbiont of Macrotermes natalensis (termites). As a consequence of this antagonism, termites produced antifungal compounds that inhibited *Trichoderma* growth (22). Likewise, fungi of the genus *Trichoderma* are plant endophytes used by the leaf-cutting ants Atta sexdens *rubropilosa* to feed their symbiont, *Leugoagaricus* (23). These authors propose that the ants can serve as a Trojan horse for *Trichoderma* spp. because these fungi use the ants as dispersers, but at the same time, they can kill the ants’ symbiont. In this sense, ants defend their symbiont by feeding on *Trichoderma* (23). Additionally, Sabatini et al. (24), to increase efficacy in the control of Gaeumannomyces graminis var. *tricini*, analyzed combinatorial effects of Trichoderma harzianum and the collembolan Onychiurus armatus (now called Orthonychiurus armatus) on the pathogen. This combination was no better than the collembolan alone in controlling the pathogen. Instead, they observed a decrease of mycelium of T. harzianum, arguing that probably the collembolan fed on it (24). Because *Trichoderma* predation by insects has not been directly studied, it remains unknown whether this fungus activates a defense response against predators.

Recently, Hernández-Oñate (25) and coworkers described the morphological and transcriptomic responses of Trichoderma atroviride to mechanical injury (MI). They observed that when a hypha is damaged, it can regenerate, producing asexual reproduction structures (conidia) in the injured area. Later, Medina-Castellanos and coworkers (26, 70) found that the signaling pathways involved in the regulation of the response involve the mitogen-activated protein kinases (MAPKs) TMK1 and TMK3. While strains with mutations in either of the corresponding genes do not produce spores in response to injury, the production and role of metabolites in the response to mechanical injury in T. atroviride have not been described. Considering the observed response of T. atroviride to injury, we propose that in nature, it could be involved in the defense against fungivorous insects.

Here, we make use of the interaction between D. melanogaster larvae and T. atroviride as a model that aims at understanding the mechanistic principles involved in fungal responses to insect attack. We show that *Drosophila* larvae do feed on T. atroviride mycelia and that the fungus responds to the attack by producing a series of metabolites. We further show that *Trichoderma* strains with mutations affecting the MAPK Tmk3 show alterations in this response, as well as in the genetic mechanisms likely involved in the activation of the chemical response to fungivory.

## RESULTS

### *Drosophila* larvae feed on *Trichoderma*.

**FIG 1:**
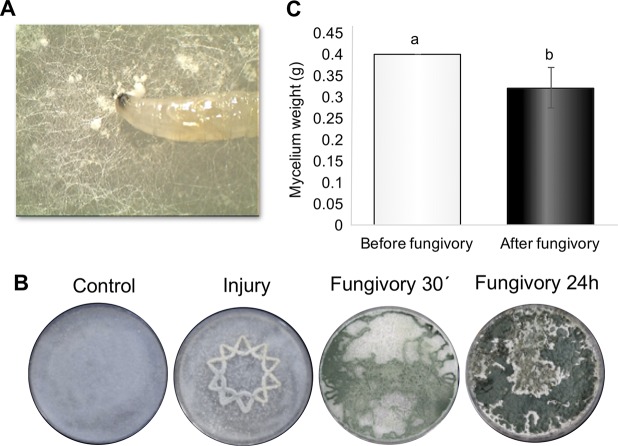
Fungivory by D. melanogaster larvae induces conidiation in T. atroviride mycelium. (A) Photograph of D. melanogaster larva feeding on T. atroviride mycelium. (B) The photographs show responses of T. atroviride to mechanical damage (Injury) and at 30 min and 24 h after fungivory, as well as an untreated control. The Control and Injury images show the response 48 h after treatment. (C) Impact of fungivory on mycelial weight. The graph shows the mycelial weights of colonies where *Drosophila* larvae grazed for 30 min. Error bar shows standard error value, and different letters indicate significant differences (*P* < 0.05).

To determine if D. melanogaster larvae, as generalist feeders, could feed on T. atroviride, we set up a simple interaction experiment, placing larvae on colonies of this fungus and allowing them to feed on mycelia for 30 min and 24 h (Fig. 1A). As shown by the results in Fig. 1B, 48 h after fungivory, all along the paths followed by larvae, formation of mature green conidia was observed. We then captured the behavior of the larvae on mycelium on video. As shown in Movie S1 in the supplemental material, D. melanogaster larvae pull and chew the mycelium, causing mechanical injury. To provide evidence that larvae indeed feed on *Trichoderma*, we weighed the mycelium of the fungus before and 4 h after placing the larvae on the mycelial mat. As shown by the results in Fig. 1C, larvae consumed 50% of the mycelium. Furthermore, we observed emergence of the fungus from surface-disinfected larval bodies (Fig. S1). Thus, in this interaction, the predator (*Trichoderma*) becomes prey.

### TMK3 mutants are more susceptible than the WT strain to fungivory by *Drosophila* larvae.

**FIG 2:**
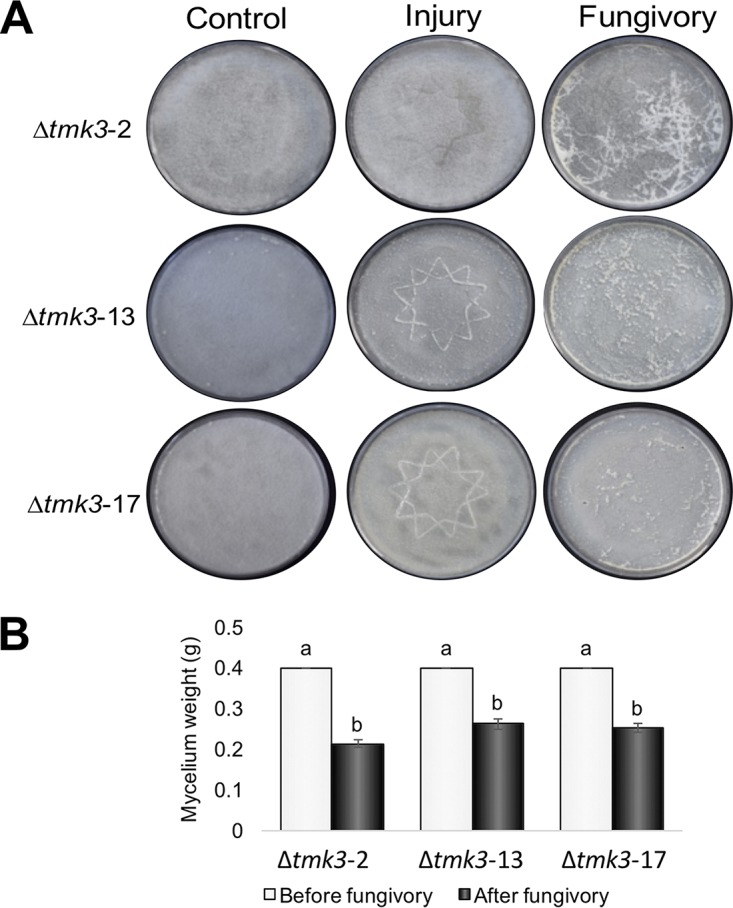
T. atroviride strains with mutations in the MAPK Tmk3 are highly susceptible to predation by *Drosophila* larvae. (A) The photographs show the response of the T. atroviride Δ*tmk3* mutants Δ*tmk3*-2, Δ*tmk3*-13, and Δ*tmk3*-17 to mechanical damage (Injury) and fungivory, as well as an untreated control. Images show the response 48 h after treatment. (B) Impact of fungivory on mycelial weight. The graph shows the mycelial weights of colonies where *Drosophila* larvae grazed for 30 min. Error bars show standard error values, and different letters indicate significant differences (*P* < 0.05).

Given that Tmk3 mutants are affected in their capacity to produce conidia in response to mechanical injury, we examined the effect of the mutation on the corresponding gene in the response to fungivory by D. melanogaster. We therefore set up *Drosophila-Trichoderma* interaction experiments using three independent Δ*tmk3* mutant strains (designated Δ*tmk3*-2, Δ*tmk3*-13, and Δ*tmk3*-17) and compared their responses to that of the wild type. As in the case of mechanical injury, the Δ*tmk3* mutants did not produce conidia in response to attack by *Drosophila* (Fig. 2A). However, the Δ*tmk3* mutants were more susceptible to predation than the WT, since the larvae consumed most of their mycelium (Fig. 2A). We thus determined mycelium weight before and 4 h after fungivory for both the WT and the Δ*tmk3* mutant strains. While the larvae consumed almost 25% (Fig. 1C) of the mycelium of the WT strain, they consumed 37 to 50% of the mycelium of the Δ*tmk3* mutant strains (Fig. 2B). Based on these results, we hypothesized that the Δ*tmk3* mutants may be affected in the production of metabolites involved in protection against or repulsion of the larvae. Given that the three mutants tested appear to respond in the same way, we decided to use only one of them (Δ*tmk3*-2) in further experiments, and we refer to it simply as Δ*tmk3*.

### Tmk3 regulates production of secondary metabolites in T. atroviride.

**FIG 3:**
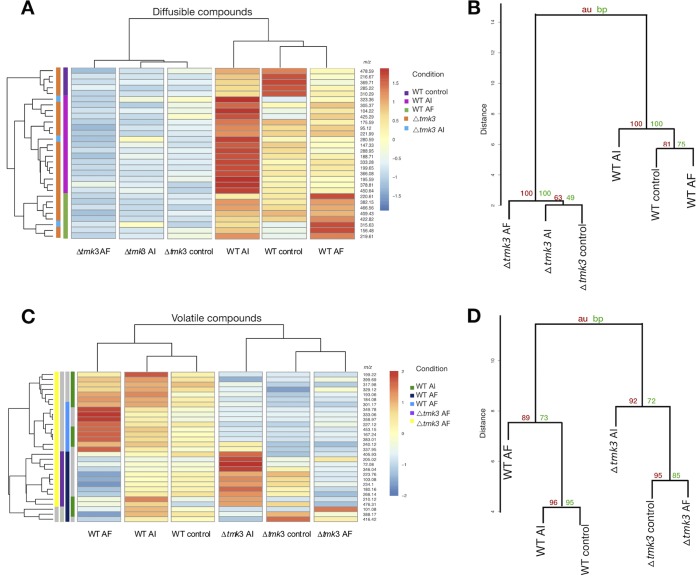
The TMK3 pathway regulates the production of secondary metabolites in response to injury and fungivory in T. atroviride. (A) Heat map of diffusible compounds produced by the WT and the Δ*tmk3* mutant after injury (AI) and after fungivory (AF). Color bars at the left side of the heat map indicate the clusters of compounds differentially produced by the WT (purple, pink, and green bars) and the Δ*tmk3* mutant (orange and blue bars). Scale bar at the right side indicates ion intensity. (B) Dendrogram that supports the clusters formed in the heat map. Numbers in red indicate the approximately unbiased (au) values, and numbers in green indicate the bootstrap probability (bp). (C) Heat map of volatile compounds differentially produced in the WT and the Δ*tmk3* mutant after injury and fungivory. Color bars at the left side of the heat map indicate the clusters of compounds differentially produced by the WT (green, dark blue, and blue bars) and the Δ*tmk3* mutant (purple and yellow bars). Scale bar at the right side indicates ion intensity. (D) Dendrogram supporting clusters formed in the heat map. Numbers in red indicate the approximately unbiased values, and numbers in green indicate the bootstrap probability.

To identify compounds that could be involved in protection against or repulsion of the larvae, we carried out metabolic fingerprinting on extracts from the Δ*tmk3* and WT strains upon mechanical injury and fungivory and from an undamaged control. We identified the 30 diffusible compounds which best explained the differences observed between the fungal strains and conditions. When we analyzed the metabolic changes in the Δ*tmk3* and WT strains in response to injury and fungivory, we observed clear differences between the patterns of diffusible compounds produced (Fig. 3A). In the control, the WT strain produced several ions but at low levels, except for a group of 5 ions that were strongly produced under this condition (Fig. 3A, purple bar at left). However, after injury, the levels of a group of 17 ions increased strongly and the levels of the compounds strongly produced under the control conditions decreased (Fig. 3A, pink bar at left). In contrast, after fungivory, the abundance of a specific group of eight ions increased even more than after injury (Fig. 3A, green bar at left). Overall, the Δ*tmk3* mutant’s production of these compounds was much lower than that of the wild type, regardless of the condition (Fig. 3A, orange bars at left). Interestingly, the production of this set of metabolites was even lower in response to fungivory; this was particularly evident in the case of the 323, 280, and 315 *m/z* ions, which increased slightly after injury (Fig. 3A, blue bars at left). A dendrogram constructed based on the patterns of metabolites produced by the two strains under the different conditions clearly shows that, although there are differential responses to injury and fungivory in both strains, the main difference in the production of metabolites is due to mutation of *tmk3* (Fig. 3B).

The production of volatile organic compounds (VOCs) was determined using a low-temperature plasma, which allows the detection of compounds of low molecular weight. VOCs produced in the WT show a medium to high intensity under control conditions. Three clusters of ions, however, increased specifically after injury in the WT strain (Fig. 3C, green bars to the left). Among these ions, we found signals indicating the presence of 6-penthyl-alpha-pyrone (6PP) at ∼167 and ∼184 *m/z* (oxygenated 6PP) (Fig. 3C), the oxygenated molecule being more abundant than in the control. Interestingly, predation by larvae resulted in decreased abundance of a large cluster of ions that were produced under the control conditions and after injury (Fig. 3C, dark-blue bar to the left). At the same time, however, fungivory greatly increased the production of a cluster of nine ions, among which we found the nonoxygenated 6PP (Fig. 3C, blue bar to the left). In the case of the Δ*tmk3* mutant, there was a set of volatile compounds that was strongly produced upon mechanical injury and detected with lower intensities in the control and at much lower intensities in the samples subjected to fungivory (Fig. 3C, purple bar to the left). It is important to notice that fungivory resulted in reduced production of most ions in the Δ*tmk3* mutant (Fig. 3C, yellow bar to the left). As in the case of diffusible compounds, the main difference in the patterns of VOC production under all conditions tested was due to a mutation of *tmk3* (Fig. 3D). The difference between the patterns of VOCs produced in response to fungivory and injury was more marked than that observed for diffusible molecules. We therefore decided to identify and compare individually the levels of production of 6PP, C_8_ compounds, and sesquiterpenes (including cyclonerodiol).

**FIG 4:**
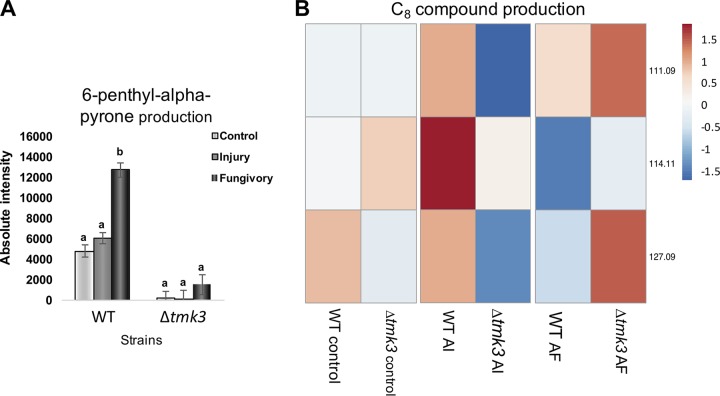
Tmk3 is involved in the production of VOCs putatively related to fungal defense. (A) Comparison of induction of 6-penthyl-alpha-pyrone production by fungivory and injury and under control conditions in WT strain and Δ*tmk3* mutant. (B) Comparison of C_8_ compound production in the WT and the Δ*tmk3* mutant after injury (AI) and after fungivory (AF). Shades of red show increases in abundance. Shades of blue represent reductions in production. Production is expressed as absolute intensity. Error bars show the standard error values, and different letters indicate significant differences (*P* < 0.05).

Fungivory, but not mechanical injury, resulted in increased production of 6PP and C_8_ compounds in the WT (Fig. 4). In contrast with 6PP, C_8_ compounds decreased and sesquiterpenes were produced at lower levels after fungivory in the WT strain (Fig. 4B; Fig. S2). Additionally, the Δ*tmk3* mutant was severely affected in the production of 6PP independently of the condition tested (Fig. 4A). While C_8_ compound production in this mutant decreased upon injury, these compounds increased after fungivory (Fig. 4B). Interestingly, the Δ*tmk3* mutant produced more sesquiterpenes than the WT upon injury (Fig. S2).

### Injury and fungivory differentially induce the expression of genes putatively involved in secondary metabolism.

To investigate whether the differential response observed in secondary metabolite production could be related to underlying gene expression, we selected four orthologs of genes that in A. nidulans are involved in secondary metabolism biosynthesis. Using bidirectional BLAST, we identified T. atroviride orthologs of the A. nidulans genes *napA* (*TanapA*; JGI gene identification number 39837), *metR* (*TametR*; JGI gene identification number 314604), and *laeA* (*Talae1*; JGI gene identification number 319344).

**FIG 5:**
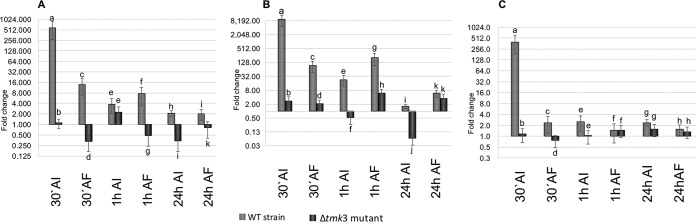
Tmk3 regulates expression of genes putatively involved in regulating transcription of genes related to secondary metabolite production in response to injury and fungivory. The graph shows the levels of messengers detected by RT-qPCR of the genes *Talae1* (A), *TametR* (B), and *TanapA* (C) at the indicated times after injury (AI) and after fungivory (AF) in the WT (gray) and the Δ*tmk3* mutant (black). The *y* axes are in log_2_. Error bars show the standard error values, and different letters indicate significant differences (*P* < 0.05).

Reverse transcription-quantitative PCR (RT-qPCR) showed that the T. atroviride ortholog of *laeA* (*Talae1*) was highly induced 30 min after mechanical injury, 575-fold, dropping to 4- and 2-fold by 1 and 24 h after injury (Fig. 5A). Interestingly, fungivory induced *Talae1* expression, but at 40-times-lower levels than mechanical injury after 30 min (14-fold) (Fig. 5A), while at 1 and 24 h, its level of expression was similar to that observed upon mechanical injury (Fig. 5A). Surprisingly, the expression of *Talae1* was repressed in the Δ*tmk3* mutant at all time points evaluated, regardless of the treatment (Fig. 5A), except for 1 h after injury, when it showed a slight induction. Similar behavior was observed for the T. atroviride ortholog of *metR* (*TametR*), which showed very strong induction (9,000-fold) upon mechanical injury in the WT strain (Fig. 5B), while after fungivory, the induction was much lower (94-fold) (Fig. 5B). In the Δ*tmk3* mutant, this gene showed very low induction early (30 min) after mechanical injury and was repressed by 1 h after the treatment, while it was slightly induced after fungivory (Fig. 5B). However, in the case of the T. atroviride ortholog of the transcription factor *napA* (*TanapA*), we observed strong induction (398-fold) in the WT 30 min after MI, which decreased dramatically (to 2-fold) 1 h after treatment and was only slightly induced during fungivory. In the Δ*tmk3* mutant, this gene was repressed early after mechanical injury, with a slight but significant induction (0.5-fold) at 24 h (Fig. 5B), whereas in response to fungivory, the expression of this gene increased very slightly (1.13- to 1.6-fold) at all time points tested (Fig. 5C).

### Metabolite production induced by injury in the Δ*tmk3* mutant make it more attractive than the WT strain to D. melanogaster larvae.

**FIG 6:**
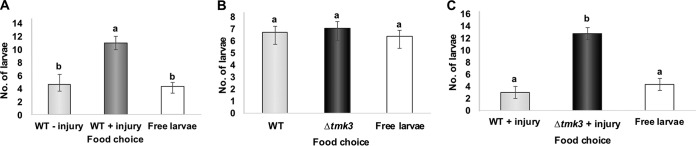
Injury induces production of compounds in T. atroviride that influence larval food choice. (A) Food choice assay between the undamaged (light gray bar) and damaged (dark gray bar) WT strain. (B) Food choice assay between the undamaged WT strain (light gray bar) and Δ*tmk3* mutant (dark gray bar). (C) Food choice assay between the damaged WT and Δ*tmk3* strains. The bars show the number of larvae that chose each of the colonies, as indicated. “Free larvae” refers to larvae that that did not choose either of the strains. Error bars show standard error values, and different letters indicate significant differences (*P* < 0.05).

Given that the different patterns of metabolite production detected between the WT and the Δ*tmk3* strains could affect their interaction with the *Drosophila* larvae, we conducted food choice assays. First, we tested the effects of mechanical injury of the WT strain on the preference of the larvae. As shown by the results in Fig. 6A, larvae preferred the damaged fungus, maybe due to the stimulation of VOC production resulting from injury (Movie S2). When examining the preference of the larvae for the WT or the Δ*tmk3* mutant without damage, we observed no difference (Fig. 6B). However, when performing the food choice assays using the mechanically injured WT and Δ*tmk3* strains, we observed that larvae had a much stronger preference for the damaged mutant than for the WT (Fig. 6C; Movie S3).

### Feeding on *Trichoderma* strongly affects development and survival of D. melanogaster larvae.

**FIG 7:**
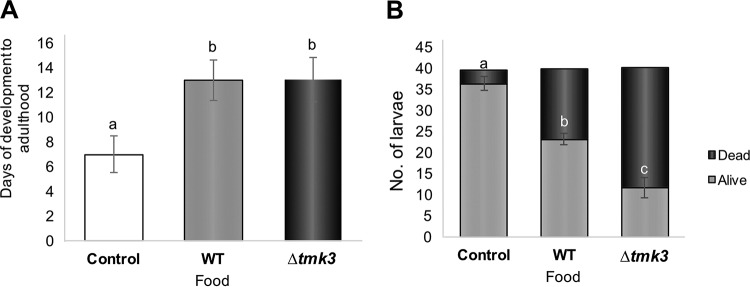
Fungal consumption slows the development of D. melanogaster larvae, while the Δ*tmk3* mutant kills a major number of larvae compared with the WT strain. (A) Larval development during 14 days after fungal consumption. Graph shows that both fungal strains affected development of larvae into adult flies. (B) Graph shows the numbers of dead larvae according to consumption of WT or Δ*tmk3* strain mycelium. Black bars show the numbers of live larvae and gray bars the numbers of dead larvae after mycelium consumption. Error bars show standard error values, and different letters indicate significant differences (*P* < 0.05).

To determine if the interaction with *Trichoderma* had any detrimental effect on the larvae, we determined larval mortality and development after feeding on the fungus. For this purpose, we allowed third-instar larvae to feed for 24 h on mycelia of the two *Trichoderma* strains and then followed their development for up to 14 days. As shown by the results in Fig. 7A, consumption of either fungal strain (WT or Δ*tmk3*) resulted in delayed development of the flies, which took ≈13 days to reach adulthood, compared to the 3 to 4 days required by the controls. While a considerable number of larvae died after interaction with the fungus, mortality was much higher when feeding on the Δ*tmk3* mutant (75%) than upon consumption of the WT (37.5%) (Fig. 7B).

### The immune system of D. melanogaster is not activated upon fungivory of *Trichoderma*.

D. melanogaster flies activate their immune system to produce antimicrobial peptides in response to attack by potentially pathogenic microorganisms. Due to the fact that *Trichoderma* consumption affects larval development and causes larval death, we considered the possibility of *Trichoderma* behaving as a pathogen of D. melanogaster. Accordingly, we proposed that antimicrobial peptide production, involved in *Drosophila*’s defense against fungi, could be affected by this interaction. To explain the high larval mortality and why one fungal genotype is more lethal, we determined the expression levels of D. melanogaster immune response genes. We asked whether differences in D. melanogaster larval mortality could be explained by differences in the recognition or production of antifungal compounds. Thus, based on the literature, we selected the genes involved in diptericin (*dptB*; FlyBase number CG10794) (67) and drosomycin (*drs*; FlyBase number CG10810) production (67) and *toll1* (FlyBase number CG5490) to determine their expression patterns in the *Trichoderma-Drosophila* interaction by RT-qPCR.

**FIG 8:**
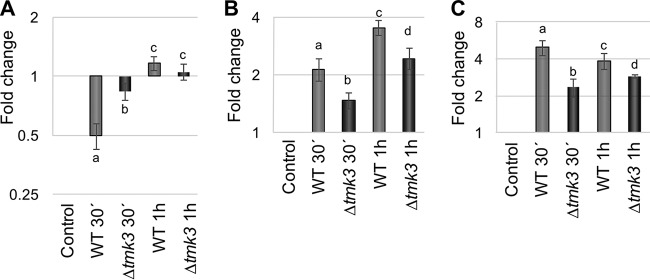
Analysis of the expression of D. melanogaster genes related to immune response to fungal attack. The graphs show the levels of messengers detected by RT-qPCR of the genes *toll1* (A), *drs1* (B), and *dptB* (C) at the indicated times after consumption of mycelium of T. atroviride WT (light gray) and the Δ*tmk3* mutant (dark gray). Error bars show the standard error values, and different letters indicate significant differences (*P* < 0.05).

Figure 8 shows the expression patterns of genes related to antimicrobial response in D. melanogaster. Expression of the Toll1 receptor-encoding gene is not activated after fungivory in any of the T. atroviride strains. In fact, the *Trichoderma* WT strain represses *toll1* expression (Fig. 8A). Interestingly, the effect of the interaction on *toll1* expression in the Δ*tmk3* mutant strain is almost negligible (Fig. 8A). Whereas expression of the genes *dptB* and *drs* was activated in both *Trichoderma* strains during fungivory (Fig. 8B and C), predation on the Δ*tmk3* strain activated them to a slightly lesser extent (Fig. 8B and C).

## DISCUSSION

Our group has previously described the early response of T. atroviride to MI (25, 26) and the role of damage-associated molecular patterns and signaling pathways (MAPKs) in the activation of the response (70). Those observations suggested that, in nature, T. atroviride uses this response to protect itself and recover from attack by fungivorous insects. Here, we show that D. melanogaster larvae feed on *Trichoderma* mycelium and that the damage caused by chewing of the mycelium induces the production of conidia in the damaged area, as previously described for mechanical injury (25). Interestingly, in some areas of the plate predated by larvae for 24 h, we observed no T. atroviride regrowth, suggesting that the larvae may produce compounds that inhibit fungal growth, such as antimicrobial peptides (28, 29).

Caballero Ortiz and coworkers (7) analyzed the expression of genes involved in protein kinase A (PKA) and MAPK signaling in A. nidulans after larval grazing (D. melanogaster larvae) and found that a set of selected genes (*pkaA*, *mpkA*, *mpkB*, and *hogA*) was upregulated in this fungus in response to fungivory. Accordingly, they postulated that MAPK and PKA signaling pathways are essential for the induction of resistance or defense against predators. In agreement with their observations, when we used a MAPK mutant (the Δ*tmk3* strain; *tmk3* is the T. atroviride ortholog of *hogA* of A. nidulans) that does not produce conidia in response to MI (26), in addition to its incapacity to conidiate in response to injury, this mutant was more susceptible to fungivory than the WT strain. Accordingly, we also propose that Tmk3 is essential to the response to fungivory in T. atroviride.

Fungi are microorganisms that produce abundant secondary metabolites (SM) (30). These secondary metabolites have been associated with communication (31, 32), development (33–35), the injury response (15, 16), and recently, a chemical response against predators (7, 11, 36–38). Metabolic fingerprinting of T. atroviride in response to injury and fungivory indicated that Tmk3 plays a major role in the regulation of metabolite production. In this regard, mechanical injury in plants induces rapid production of VOCs that can serve to protect the plant from future attack by herbivorous insects (39–42). In fungi, however, studies on the metabolic response to injury have focused on fruiting bodies of basidiomycetes, where, after injury, inactive precursors are enzymatically transformed into active compounds with antimicrobial or toxic properties (15, 16). Our results show that, in response to mechanical injury, filamentous fungi rapidly produce VOCs that could be the result of wound-activated or wound-induced chemical defense (15, 16). On the other hand, herbivores can suppress the induction of jasmonic acid (JA)- and salicylic acid (SA)-dependent direct and indirect defenses in plants (42, 43) and reduce the production or accumulation of plant defense compounds (43, 44). As shown in our metabolic profiling, fungivory results in changes in the production of metabolites and reduces or increases the abundance of specific ions in T. atroviride. Based on these observations, we hypothesize that *Drosophila* larval saliva contains a molecule(s) that provoke(s) specific changes in the fungal metabolite profile related to defense or resistance, as suggested previously (45, 46).

Exogenous VOCs, such C_8_ compounds, have been correlated with the activation of conidiation in *Trichoderma* spp. (47). However, the mechanism involved in the specific response to C_8_ compounds has not been determined. Furthermore, G protein or MAPK pathways have been suggested to mediate perception of C_8_ compounds (48). C_8_ compound production in the Δ*tmk3* strain is reduced upon injury compared with that in the WT, suggesting that this pathway may be involved in the production of C_8_ compounds after mechanical damage and that this defect might explain the strongly reduced production of conidia by this mutant (26). Thus, the TMK3 pathway could activate C_8_ compound production in response to injury and lead to chemical protection and survival through the production of conidia. In relation to the chemical defense of fungi against predators, C_8_ compounds are known to have a neurotoxic effect that impairs *Drosophila* larval development (49) and to act as deterrents of fungivorous insects (50). In this sense, we showed that after fungivory, the production of C_8_ compounds is higher in the Δ*tmk3* mutant than in the WT, which might explain the amount of larval death observed upon feeding on the mutant.

Previous reports of the response of fungi to biotic and abiotic stress have shown that fungi can trigger specific transcriptional responses, depending on the type of stress (9, 25, 51, 52). However, comparative studies of the transcriptional responses to injury and fungivory are scarce. Here, we describe the differential gene expression of T. atroviride to mechanical injury and fungivory and the importance of the MAPK Tmk3 in mediating the activation of genes related to these responses. Our results show that fungivory reduces the transcript levels of *Talae1*, *TametR*, and *TanapA*, the genes selected for study, in the WT strain. It is plausible that the saliva of *Drosophila* larvae contains compounds that interfere with the transcriptional activation of these genes. Furthermore, the expression of this set of genes is either repressed or very poorly activated in the Δ*tmk3* mutant, regardless of the treatment. The lack of activation of genes potentially involved in chemical defense might explain why the Δ*tmk3* mutant is more susceptible than the wild-type strain to predation by *Drosophila* larvae.

D. melanogaster flies can detect fungi in nature (53) and sense the metabolites produced by them (6, 7). As described here, injury causes metabolic changes in T. atroviride that could represent a chemical defense mechanism. In food choice assays, however, larvae preferred damaged to undamaged fungi. In this sense, many fungal volatiles, particularly 8-carbon alcohols and ketones, act as semiochemicals and function in insect attraction and deterrence (54). Thus, it is likely that the compounds produced after injury could serve as larval attractants. Interestingly, larvae prefer the damaged Δ*tmk3* mutant rather than the damaged WT strain. In this regard, according to our VOC profiling, injury results in increases in the abundance of a group of ions in the Δ*tmk3* mutant that are not present in the WT or in the Δ*tmk3* strain after fungivory. These compounds could be more attractive than those produced by the WT strain after injury. An alternative explanation is that the Δ*tmk3* strain does not produce some ions that serve as insect repellents. Several authors have reported that mutants affected in secondary metabolite production are more attractive to fungivorous insects, such as D. melanogaster larvae, or collembolans (8, 11, 13, 14). These authors also described the effects of fungal SM in larval development (6–8). Similarly, we observed that T. atroviride consumption delays larva-to-adult fly development and a large number of larvae die. Nevertheless, our results show that, in our case, the mutant affected in metabolite production kills more larvae than the WT strain. Probably high consumption of the Δ*tmk3* mycelium is more toxic to the larvae than consumption of the WT mycelium. On the other hand, antimicrobial peptide (AMP) production is one of the principal barriers of the immune system of *Drosophila* against antagonistic microorganisms (55). Here, we show that both *Trichoderma* strains repress the expression of the *toll1* receptor-encoding gene but, at the same time, activate the expression of the genes *dptB* and *drs* (encoding diptericin and drosomycin). These results suggest that *Trichoderma* does not activate the production of drosomycin through the Toll1 pathway but through activation of the Toll5 pathway, which has also been reported to activate drosomycin production (56, 57). The delayed larval development observed may reflect repression of the Toll1 pathway, as Toll1 is involved in *Drosophila*’s development (58, 59). Furthermore, repression of *toll1* after fungivory may affect the expression of other genes related to AMP production and defense, such as cecropin, attacin, and defensin (57), causing larval mortality. While the *dptB* gene is activated primarily by the confrontation of *Drosophila* with Gram-negative bacteria, filamentous fungi can also activate *dptB* gene expression at lower levels (57). In our study, both *Trichoderma* strains induced *dptB* gene expression, at even higher levels than the *drs* gene.

In conclusion, we showed that T. atroviride differentially regulates its metabolic responses to mechanical injury and fungivory. Furthermore, we showed that the TMK3 pathway plays an important role in the regulation of T. atroviride’s metabolite production, as the mutation in this gene affects the production of compounds putatively involved in fungal defense and resistance, such as C_8_ compounds, 6-penthyl-alpha-pyrone, and sesquiterpenes. Tmk3 represents at least one signaling pathway that triggers the transcriptional activation of a genetic mechanism necessary to respond to predators. The TMK3 pathway could be regulating the T. atroviride response to fungivory through an epigenetic mechanism mediated by the methyltransferase Lae1 and a transcriptional-activation process mediated by bZIP-type transcription factors. Future studies should address the probably specific genetic regulation of injury and fungivory responses and how secondary metabolite production is specifically activated at the genetic level.

## MATERIALS AND METHODS

### Biological material and growth conditions. (i) Fungal strains.

Trichoderma atroviride strain IMI 2060040 was used as the wild type (WT). The T. atroviride mutants Δ*tmk3*-2, Δ*tmk3*-13, and Δ*tmk3*-17 were previously described (60). All strains were propagated on potato dextrose agar (PDA; Difco) at 39 g/liter in constant light at 27°C for 5 days.

### (ii) Insects and larvae.

The founder of the isofemale strain Drosophila melanogaster SD-5 was collected in San Diego, CA, USA. Adult flies were preserved on standard banana medium with a photoperiod of 12 h dark/12 h light at 25°C. For egg collection, 300 flies were placed into a collector with a petri dish containing flour-agar medium (10 g/liter agar, 25 g/liter sucrose, 105.5 g/liter wheat flour, and 18 g/liter Bacto yeast extract). Eggs were incubated in flour-agar medium for 5 days to obtain third-instar larvae. Larvae were collected using dissection forceps and washed three times with sterile 1× phosphate-buffered saline (PBS) (8 g/liter NaCl, 0.2 g/liter KCl, 1.44 g/liter Na_2_HPO_4_, 0.24 g/liter KH_2_PO_4_, pH adjusted to 7.4 with HCl).

### Interactions between D. melanogaster and T. atroviride.

For interaction experiments, spores collected from a fresh culture of T. atroviride (5 days of incubation) were counted in a Newbauer chamber, and 10,000 were dispersed on a petri dish containing PDA and incubated for 36 h at 27°C in darkness. Forty larvae were then placed on the mycelial fungal mat of the fungus and allowed to feed for 30 min, 1 h, or 24 h. Larvae were placed and removed with dissection forceps under red light to avoid photoinduction of conidiation. Fungi were incubated for an additional 48-h period at 26°C in darkness. As controls, we used fungal colonies injured with a sterile scalpel and untreated colonies of the same age.

### *Trichoderma* survival in the larvae.

To confirm consumption of *Trichoderma* by larvae, we collected the larval bodies after they had fed on the mycelia of the WT and the *tmk3* mutants. Larvae were washed two times with ethanol (70%), once with sodium hypochlorite (20%), and three times with distilled sterile water to eliminate any living fungal structures from outside the body. Larvae were then frozen at −20°C for 2 h and placed in *Trichoderma* selective culture medium (rose Bengal medium containing 0.2 g/liter MgSO_4_·7H_2_O, 0.9 g/liter K_2_HPO_4_, 0.15 g/liter KCl, 1 g/liter NH_4_NO_3_, 3 g/liter glucose, 0.075 g/liter rose Bengal, and 20 g/liter agar, which was sterilized by heat for 20 min at 120°C before 0.25 g/liter chloramphenicol and 0.2 g/liter quintozene were added). Plates were incubated in darkness for 3 days at 27°C. After 3 days, larval bodies from which fungal colonies emerged were counted. We used triplicates of 40 larvae in three independent experiments for statistical analysis.

### Mycelial weight loss analysis.

To determine the effect of the larvae on the mycelia, interaction experiments were performed in petri dishes containing PDA medium covered with sterile cellophane sheets (8 cm in diameter, heat sterilized). Four hundred milligrams of mycelium of each strain was used to feed larvae. The mycelium was placed in the middle of the plate, and then 40 larvae were placed on the mycelium. Four hours later, the mycelium was weighed in an analytical balance. For statistical analyses, we used three replicates in three independent experiments.

### Metabolic fingerprinting by direct mass spectrometry.

Metabolic fingerprinting of T. atroviride after injury and fungivory was conducted using two techniques: direct liquid injection mass spectrometry (DLI-MS) and low-temperature plasma (LTP) ionization mass spectrometry (LTP-MS).

For the DLI-MS analysis, we obtained extracts based on a previously reported methodology (61), with some modifications based on the protocol reported by Frisvad (62), as follows: 100 mg of mycelium was homogenized and extracted with high-performance liquid chromatography (HPLC)-grade ethyl acetate, methanol, and dichloromethane with proportions of 3:2:1, respectively, and 1% formic acid (Sigma-Aldrich, Mexico). The sample was then disrupted using a sonicator (Branson 1510) at a frequency of 40 kHz for 50 min at room temperature, after which the solvent was evaporated with nitrogen gas. Residual compounds were resuspended in 400 μl of HPLC-grade methanol (Fisher Scientific, Mexico) and filtered through a 0.2-μm nylon membrane (Thermo Scientific, Mexico). Mass spectra were acquired in positive ESI mode, using an LCQ Fleet ion trap mass spectrometer (Thermo Scientific, USA).

For the measurements, the capillary voltage was set to 35 V, the capillary temperature to 280°C, the tube lens to 80 V, and the spray voltage to 4.5 kV. Spectra were acquired in positive profile mode, in an *m/z* range of 50 to 500.

For the direct analysis with LTP-MS, we used a device designed in the laboratory (63, 64). The complete plate of T. atroviride was placed under the plasma beam; extraction of metabolites was not required. The measurements were performed in triplicates. The LTP was mounted perpendicular to the MS inlet. The technical parameters were the same as reported previously (63, 64). Spectra were acquired in positive and profile mode, in an *m/z* range of 50 to 500. The DLI-MS and LTP-MS analyses were carried out in three independent experiments.

### Metabolic data processing and statistical analyses.

The raw mass spectrometry data were transformed to the HUPO standard data format, mzML, using msconvert version 3.0.91 (65). The spectra then were processed using a workflow designed in R (https://www.r-project.org), employing the package MALDIquant (65). All scans of a sample were summarized and smoothed using a Savitzky-Golay filter. Peaks were detected with a signal-to-noise ratio of 2 and aligned to allow the comparison of peaks across different samples.

To identify the most important ion signals, we generated a random forest tree model with 500 tree decisions. The algorithm ranks the most important ions based on the Gini index. We used the 30 most important signals, i.e., the ions that best explain the differences between the wild-type and mutant samples. For data mining and model building, we used the R package Rattle (27) and the mean values from triplicate experiments. We applied a hierarchical clustering analysis (HCA) to the filtered data to create metabolic heat maps and evaluated differences in the metabolic profiles (66).

### RT-qPCR.

For analysis of the expression of *Trichoderma* genes, mycelia were collected from *Trichoderma-Drosophila* interaction (see “Interactions between D. melanogaster and T. atroviride,” above) or mechanical injury experiments, collected, and immediately frozen in liquid nitrogen. For analysis of the expression of *Drosophila* genes, 40 larvae were collected and used for RNA extraction. Total RNA was extracted using TRIzol, following the manufacturer’s instructions. For RT-qPCR, primers were designed to produce amplicons of around 150 bp (Table 1) (28, 67, 68) for all selected genes and for a gene encoding DNA polymerase family B as an internal control housekeeping gene. cDNA was synthesized using Superscript reverse transcriptase II (Invitrogen). The reaction mixture for quantitative PCR was as follows: 10 μl of SYBR green master mix (Applied Biosystems), 3 ml of cDNA template (3 ng/ml), and 1 μl (10 μM) of each of the primers. The PCR program was as follows: one cycle at 95°C for 5 min and 40 cycles at 95°C for 30 s, 65°C for 30 s, and 72°C for 40 s. Melting curves for each product, starting from 60°C and increasing to 95°C at 0.2°C/s, produced a single melting point. All experiments were repeated at least three times. All calculations and analyses were performed using ABI 7500 software, version 2.0.1 (Applied Biosystems), and the cycle threshold (2^−ΔΔ^*^CT^*) method (69).

**TABLE 1:** Sequences of primers used for RT-qPCR

Organism, gene	Gene identifier	Sequence	
		Forward	Reverse
*T. atroviride*			
*lae1*	319344	AACGCCCCTTTCTGTAACCT	GCGACTAGCTCATCCTGGAC
*napA*	39837	AATGGCAGGAGAAAAGCTGA	GATTAGCCAGCCACTGCTTC
*metR*	314604	GTGGACGAATACGCCAAACT	TAGCGTGCTGCCAATAACTG
DNA pol	53190	GAGTGGCGTATGTCATGGTG	GCGTCTTCTTGGCAAACTTC

*D. melanogaster*			
*toll1*	CG5490	CCCAACAAACACCTGAACG	GTCACAAGGTTGCCCAAGTT
*dsr*	CG10810	GTACTTGTTCGCCCTCTTCG	ATTTAGCATCCTTCGCACCA
*dptB*	CG10794	ATCCTGATCCCCGAGAGATT	CGTTGAGGCTCAGATCGAAT
18S rRNA	FBgn0061475	GGTCTGTGATGCCTTTAGATGTCC	GACCTCTCGGTCTAGGAAATACAC

### Food choice assays.

The food choice assays were performed in 150- by 150-mm petri dishes containing PDA. One thousand conidia of the WT strain of T. atroviride or the Δ*tmk3* mutant were inoculated at 1 cm from the end of the plate and incubated for 36 h at 26°C in darkness. One third-instar larva was placed in the middle of the plate and video recorded for 15 min. Twenty replicates were used per food choice assay, in three independent experiments. For statistical analysis, we counted the number of replicates in which larvae were at the side of the indicated fungal strain at the end of the 15-min period.

### Larval survival.

To determine the effect of *Trichoderma* consumption on the larvae, analyses of interactions between D. melanogaster larvae and T. atroviride were performed as described above in “Interactions between D. melanogaster and T. atroviride.” After 30 min of interaction of the larvae with the indicated fungal strains, the larvae were placed on fresh PDA plates and incubated until they completed their life cycle. Larval development and death were monitored every 24 h for 14 days. Once all living larvae reached adulthood, the numbers of dead and living flies were computed. As a control, we used larvae that did not feed on *Trichoderma*. The experiment was performed using three technical replicates of 40 larvae in three independent assays.

### Statistical analysis.

Single-factor analysis of variance (ANOVA) was used, followed by a Fisher’s exact test. Statistically significant differences in mean values at a *P* value of <0.05 are indicated with different letters in the figures.

## Supplementary Material

Supplemental file 1

Supplemental file 2

Supplemental file 3

Supplemental file 4
