# Supplementary material for: Trichoderma atroviride from Predator to Prey: Role of the Mitogen-Activated Protein Kinase Tmk3 in Fungal Chemical Defense against Fungivory by Drosophila melanogaster Larvae
Source: Appl Environ Microbiol. 2019 Jan 9;85(2):e01825-18. doi: 10.1128/AEM.01825-18 (PMC6328759; doi:10.1128/AEM.01825-18)
Supplement: Supplemental file 1 [file AEM.01825-18-s0001.pdf]

S1

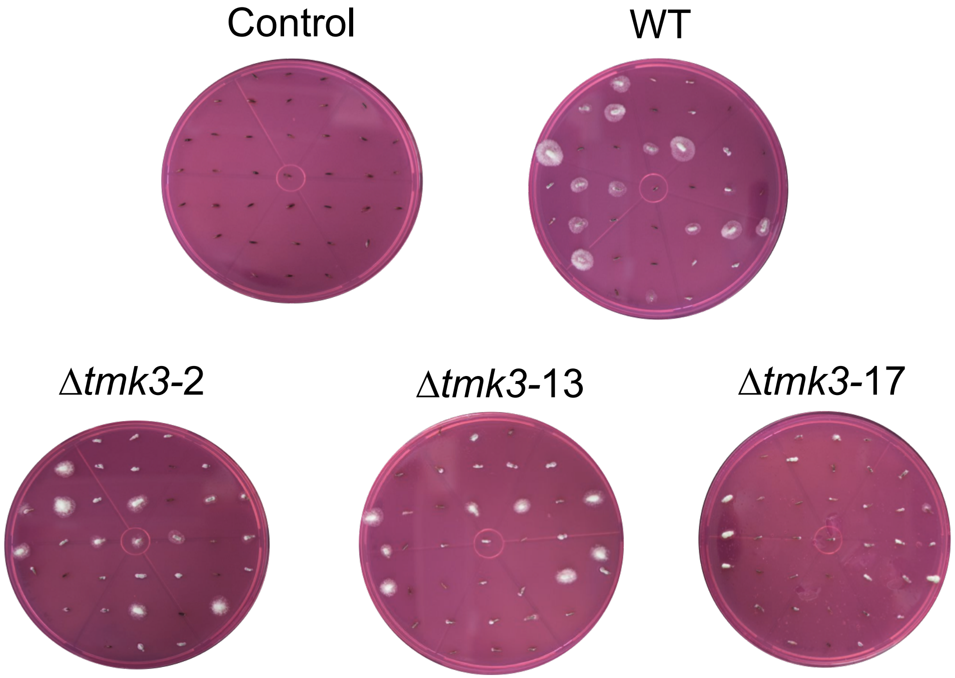

Figure S1. *Trichoderma* emerging from larval bodies. The photographs show *Trichoderma* mycelium of the indicated strain emerging from surface disinfected larval bodies and growing on selective medium.

S2

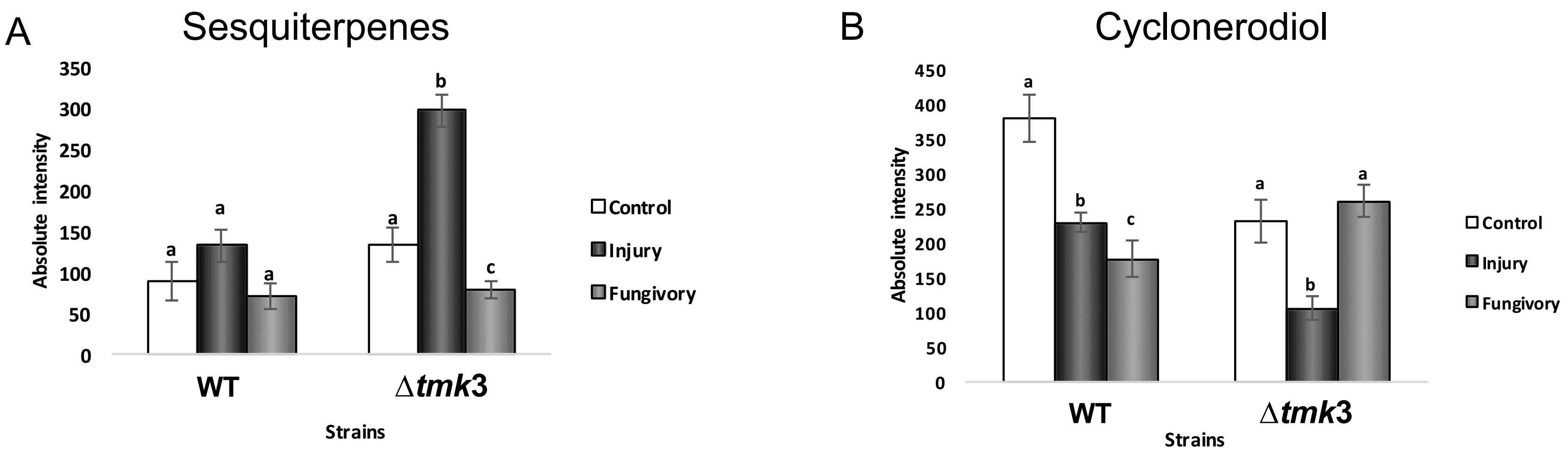

Figure S2. **Insecticidal volatile compounds production**. **A**) Comparison of the sesquiterpenes production with a similar *m/z* in the WT and Δ*tmk3* mutant after injury (black) and fungivory (gray) compared with basal production (white). **B**) comparison of the cyclonerodiol production in WT and Δ*tmk3* mutant after injury (black) and fungivory (gray) compared with basal production (white). Production is expressed in absolute intensity. Bars in graph show standard error values and different letters means significant differences (P<0-05).

Movie S1. *Drosophila* grazing on *Trichoderma*. Video showing *D. melanogaster* larvae feeding on the *T. atroviride* mycelium. It is clearly observed how the larva pulls the mycelium to eat it.

Movie S2. *Drosophila* larvae prefer feeding on damaged mycelium. The movie shows the behavior of larvae during food choice assays, where it is clear that larvae prefer feeding on the damaged than on the undamaged WT strain. The movie is 15X accelerated.

Movie S3. *Drosophila* larvae prefer feeding on a mutant affected in secondary metabolite production. Food choice assay between the damaged strains (WT and Δ*tmk*3). The movie shows larval attraction by the damaged Δ*tmk*3 mutant. The movie is 8X accelerated.
